# Ureterocoele Complicated By Cystolithiasis in a 23-Year-Old: A Case Report

**DOI:** 10.4314/ejhs.v34i3.11

**Published:** 2024-05

**Authors:** Obirija Samson Emeka, Rasheed Mumini Wemimo, LatifatTunrayo Oduola-Owoo, Okonkwo Juliet Ebele

**Affiliations:** 1 Department of Radiology, Alex Ekwueme Federal University Teaching Hospital, Abakaliki Ebonyi State; 2 Department of Anatomic Pathology, Federal University Dutse and Rasheed Shekoni Federal University Teaching Hospital, Jigawa State; 3 Department of Radiology, Federal Medical Centre, Ebutte Meta, Lagos; 4 Department of Radiology, Lagos University Teaching Hospital, Idi-Araba, Lagos State

**Keywords:** Ureterocoele, Cystolithiasis, Ultrasonography, Intravenous urography

## Abstract

The cystic dilatation of the lower part of the ureter is known as ureterocoele which has been associated with other anomalies such as stenotic ureteric orifice, duplicated urinary system, and hydronephrosis along with or without other clinical sequelae. Notably, the accurate and prompt diagnosis is challenging due to non-specific symptoms that could mimic other urogenital systems diseases. Imaging evaluation is the gold standard of accurate diagnosis and comprehensive clinical examination. The possible complications associated with ureterocoele include urinary tract obstruction, reflux, continence, and renal function derangements. Thus, we present a 23-year-old male patient, who presented to the general outpatient clinic of the Federal Teaching Hospital, Abakaliki, in September 2018 with a complaint of right-sidedabdominal pain and hot sensation, intermittent haematuria, and pain during micturition of one-year duration. He was evaluated with ultrasonography that showed moderate right hydronephrosis with a large curvilinear calculus adjacent to the dilated ureteric end, and intravenous urography revealed a dilated right distal ureteric end with a peripheral halo, giving the cobra head the appearance of ureterocoele. The patient underwent open cystolithotomy with marsupialization of the distal end of the right ureter. He showed remarkable post-operative improvement and was discharged after two weeks. Two months of post-operative follow-up was uneventful.

## Introduction

Ureterocoele is a congenital abnormality characterized by cystic dilatation of the distal end of the ureter (10). It is often associated with other abnormalities like stenotic uretericorifice, duplicated urinary system, and hydronephrosis along with or without other clinical sequelae. Complications associated withureterocoele include urinary tract obstruction, reflux, continence, and renal function derangements (1,2). Ureteroceles may be intravesical (orthotopic) or extravesical (ectopic) (2). It has a prevalence of 1 in 4000-5000 individuals with a female-to-male prevalence ratio of 4:1; it remains a diagnostic and treatment challengeowing to its different types and clinical presentations (1). This report is a case of right-sided intravesical ureterocele with a large impacted urinary bladder calculus in a 23-year-old male patient at the Federal Teaching Hospital, Abakaliki.

## Case Presentation

Mr. GP, a 23-year-old male patient, presented to the General Outpatient Clinic of the Federal Teaching Hospital, Abakaliki, in September 2018 with a complaint of right-sided abdominal pain and hot sensation, intermittent haematuria, and pain during micturition of one-year duration. The pain was steady, mild-moderate in severity, and worse on the right. There was associated haematuria. There was no history of pelvic trauma or urethral instrumentation. There was associated dysuria and a feeling of incomplete emptying of the urinary bladder. The patient had patronized many alternative medicine outfits where he was diagnosed of suffering from ‘Internal heat’ and had different concoctions administered. He had also been placed on ciprofloxacin by a patent medicine dealer one week prior to presentation. He presented to the Outpatient Clinic on account of worsening symptoms and increased haematuria. Physical examination revealed a pale-looking young man in no obvious respiratory distress. He was anicteric, afebrile to touch, acyanosed, no pedal oedema, and no lymphadenopathy.

Pulse rate was 88b/min, respiratory rate was 20c/min, blood pressure was 140/60mmHg, and temperature was 36.5°C. The kidneys were ballotable with renal angle tenderness noted on the right. There was also suprapubic tenderness. The urology team was invited to review and take over the management based on history and physical examination findings.

Full blood count showedhaemoglobin level of 10.5mg/dl;white blood count was normal for both total and differential. Urinalysis revealed haematuria, and urine microscopy, culture, and sensitivity were unremarkable. The following laboratory tests were obtained: VDRL, retroviral screening test, and HBsAg; HCV were all negative.

**Figure 1:**
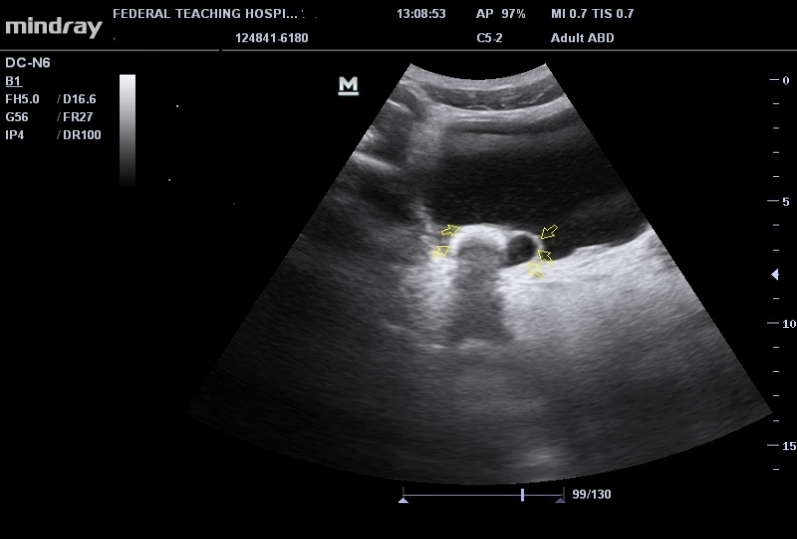
Ultrasonography of the urinary bladder showing dilated distal (intravesical) end of the right ureter giving the cyst in urinary bladder appearance, with a curvilinear urinary bladder calculus casting posterior acoustic shadowing impacted around the neck of the ureterocele (arrows)

**Figure 2:**
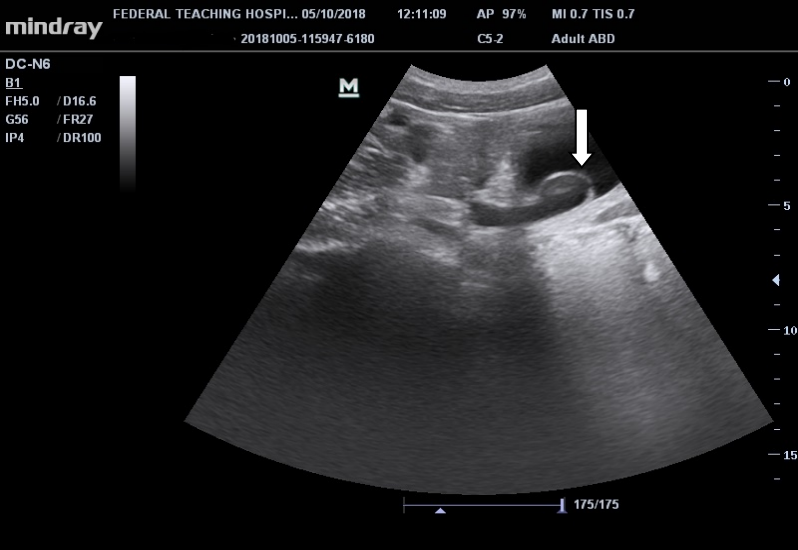
Ultrasonographic image of the urinary bladder showing dilated intravesical right ureter with cobra head sign (white arrow)

**Figure 3:**
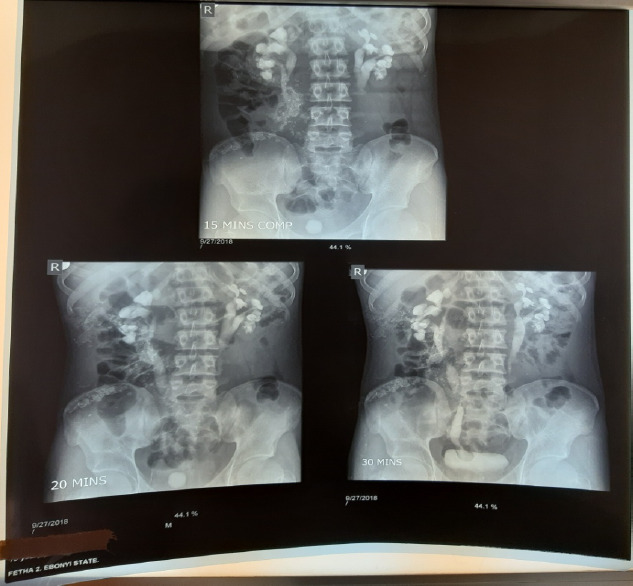
Series of intravenous urography (IVU) taken at 15,20, and minute showing urinary bladder calculus, bilateral moderate hydronephrosis, and dilated right intravesical ureter with peripheral halo-giving the classical ‘cobra head’ appearance (arrow)

Ultrasonography showed moderate right hydronephrosis with a dilated distal end of the right ureter, giving a cyst-within-the-bladder appearance and a large curvilinear calculus adjacent to the dilated ureteric end as illustrated in Figures 1 and 2. Intravenous urography revealed a dilated right distal ureteric end with a peripheral halo, giving the cobra head the appearance of ureterocoele, bilateral hydronephrosis (grade 4 on the right and 3 on the left), large rounded urinary bladder calculus as shown in Figure 3.

Based on imaging findings, a diagnosis of right Ureterocoele with urinary bladder stone (cystolithiasis) was made. The patient underwent open cystolithotomy with marsupialization of the distal end of the right ureter. He showed remarkable post-operative improvement and was discharged after two weeks. Two months of post-operative follow-up was uneventful.

The case report was conducted in compliance with the guidelines of the Helsinki Declaration on biomedical research on human subjects, and informed consent of the patient for publication was obtained.

## Discussion

Ureterocoele is a cystic dilatation of the distal end of the ureter that may be intravesical or extravesical (2). It is a urological developmental anomaly in which exact pathogenesis has remained controversial. Among the plausible theories propounded on the pathogenesis of ureterocoeleis abnormal muscular development of the distal ureter with associated weakness and dilatation; abnormal developmental stimulus was also believed to be responsible for the dilatation (1,3).

The most appealing of thesemechanisms is that of incomplete dissolution of Chwalla's membrane which is a partition between the urogenital sinus and developing ureteral bud and is usually present before the 37th day of gestation. Persistence of this membrane may lead to weakness and dilatation of the distal ureter (3,4). It is often linked with duplex upper tract and ectopic ureteric insertions (3).

Its effects may include urinary tract obstruction, reflux, continence, and renal function derangements. The incidence of ureterocoeles is 1:4000-5000 individuals. It is 4:1 commoner in females, slightly more on the left side, with 10% of the cases seen bilaterally (5). Ureterocoeles have diverse presentations which include life-threatening sepsis, renal failure, recurrent urinary tract infections (UTIs), or maybe, asymptomatic and only detected incidentally or byantenatal ultrasonography (3).

Thus, there are variations in the clinical presentation in relation to different types of ureterocoeles. The classification systems include Stephen, Churchill, and the American Academy of Paediatrics classification. The American Academy of Paediatrics classified ureterocoele into orthotopic (intravesical) and ectopic (extravesical) (4). This classification is the most popular and has gained wide acceptance among clinicians and radiologists.

Our patient had the orthotopic (intravesical) variant. Ureterocoele is a rare disease among the non-Caucasians with an unknown incidence rate among Africans. This was captured in a study done in Zaria (2012) by Muhammed et al (5). Their study is a retrospective study of 10 patients with ureterocoele treated in Ahmadu Bello University Teaching Hospital, Zaria. In their study, the presenting symptoms are flank pain, dysuria, lower urinary tract symptoms (LUTS), fever, andhaematuria, with corresponding frequencies of 60%, 60%,50%, 40%, and 30% respectively.

They also found out that impacted stones occur in 50% of cases, and only 1 out of 10 subjects (10%) had associated duplex renal system (5).However, there was no associated impacted stone in this index patient at surgery. They also noted that unilateral and bilateral ureteroceles were found in 10% and 90% of their subjects respectively (5).

The above figures are close to 10% and 80% for unilateral and bilateral ureterocele respectively that was documented among the Caucasian population (3). The single system ureterocoele (unilateral variant) is commonly found in adults and is known as ‘adult ureterocele’. They are usually orthotopic and less prone to obstruction and renal dysplasia due to a duplicated renal system (2,3). This may explain why this index patient presented late with mild to moderate hydronephrosis (obstructive feature). There was no previous suspicion prior to imaging of this patient due to the rarity of the disease and the fact that it is predominantly an onset of a supposedly childhood disease.

Muhammed et al also noted that none of the 10 patients reviewed in their study had a prior clinical suspicion of ureterocoele before imaging (5). Some researchers documented an association between adult ureterocoele and other genitourinary diseases; and called it “acquired ureterocoele,”((2). Umerah BC (1977) and Elem et al (1981) reported cases of ureterocoele associated with schistosomiasis (5).

Muhammed et al (5) recorded the following associations among their subjects: schistosomiasis in 10%, genitourinary tuberculosis (GU TB) in 30%, and impacted stone in 30%. The index patient had an associated impacted stone, and similar findings were reported by another reviewer (5).However, this patient was not screened for either genitourinary TB (GU TB) or schistosomiasis.

More importantly, the diagnosis of ureterocele is by imaging. Abdominopelvic ultrasonography may show cyst within the urinary bladder or close to the proximal urethra [4]. Intravenous urography (IVU) may show poor function of the affected side with delayed or absent excretion of contrast, hydroureteronephrosis, dilated distal ureter, and lucent peripheral halo consistent with the classical ‘cobra head’ sign (4).

Voiding cystourethrography (VCUG) can also assess the ureter for reflux into the ureters [5]. Scintigraphy is also useful and may demonstrate renal function changes, and detect obstruction [5]. This index patient had ultrasonography and intravenous urography; and both modalities revealed classical findings for ureterocele. Thereafter, the definitive diagnosis was made based on imaging findings as demonstrated in figures 1, 2, and 3. The imaging studies demonstrate calculus within the dilated ureter. Early intervention is encouraged in diagnosed cases of ureterocoele for cure and preservation of renal function to avoid long-term complications like obstruction, infection, or reflux(5).

Treatment options can be endoscopic or open surgical treatment. The endoscopic methods include transurethral puncture and transurethral incision while the open types include intravesical excision with ureteric reimplantation, marsupialization, upper pole nephrectomy, and partial uretectomy in cases with dysplastic kidney due to duplex system (1).

The index patient underwent open cystolithotomy with marsupialization of the distal end of the right ureter. The endoscopic method is associated with a high curative rate (90%), and short hospital stay (5). However, the open method is predominantly used in our environment as noted by Muhammed et al in which 70% of their patients were treated through this method (5). This may be explained by the paucity of equipment and expertise.

Finally, CT urographywhich is an excellent technique could be used to diagnose this lesion and urinary tract stones due to high sensitivity and specificity, but the patient opted for intravenous urogram (IVU) owing to economic reasons. In conclusion,Adult ureterocoele is a rare but important urological disease that may lead to other renal and systemic complications. It is therefore important that a high index of suspicion be maintained and early imaging evaluation obtained in patients with inexplicable urinary tract symptoms, recurrent infections, dysuria, haematuria, flank pain, and fever. This is important as early diagnosis and treatment may help to avoid long-term complications.
